# Elucidating the Metabolic Plasticity of Cancer: Mitochondrial Reprogramming and Hybrid Metabolic States

**DOI:** 10.3390/cells7030021

**Published:** 2018-03-13

**Authors:** Dongya Jia, Jun Hyoung Park, Kwang Hwa Jung, Herbert Levine, Benny Abraham Kaipparettu

**Affiliations:** 1 Center for Theoretical Biological Physics, Rice University, Houston, TX 77005, USA; dyajia@gmail.com (D.J.); herbert.levine@rice.edu (H.L.); 2 Systems, Synthetic and Physical Biology Program, Rice University, Houston, TX 77005, USA; 3 Department of Molecular and Human Genetics, Baylor College of Medicine, Houston, TX 77030, USA; Junhyoung.Park@bcm.edu (J.H.P.); kwanghwa.jung@bcm.edu (K.H.J.); 4 Department of Bioengineering, Rice University, Houston, TX 77005, USA; 5 Department of Biosciences, Rice University, Houston, TX 77005, USA; 6 Physics and Astronomy, Rice University, Houston, TX 77005, USA; 7 Dan L. Duncan Comprehensive Cancer Center, Baylor College of Medicine, Houston, TX 77030, USA

**Keywords:** cancer metabolism, Warburg effect, oxidative phosphorylation, OXPHOS, mitochondrial respiration, hybrid metabolic phenotype, metabolic plasticity, tumorigenesis, metastasis, EMT, stemness

## Abstract

Aerobic glycolysis, also referred to as the Warburg effect, has been regarded as the dominant metabolic phenotype in cancer cells for a long time. More recently, it has been shown that mitochondria in most tumors are not defective in their ability to carry out oxidative phosphorylation (OXPHOS). Instead, in highly aggressive cancer cells, mitochondrial energy pathways are reprogrammed to meet the challenges of high energy demand, better utilization of available fuels and macromolecular synthesis for rapid cell division and migration. Mitochondrial energy reprogramming is also involved in the regulation of oncogenic pathways via mitochondria-to-nucleus retrograde signaling and post-translational modification of oncoproteins. In addition, neoplastic mitochondria can engage in crosstalk with the tumor microenvironment. For example, signals from cancer-associated fibroblasts can drive tumor mitochondria to utilize OXPHOS, a process known as the reverse Warburg effect. Emerging evidence shows that cancer cells can acquire a hybrid glycolysis/OXPHOS phenotype in which both glycolysis and OXPHOS can be utilized for energy production and biomass synthesis. The hybrid glycolysis/OXPHOS phenotype facilitates metabolic plasticity of cancer cells and may be specifically associated with metastasis and therapy-resistance. Moreover, cancer cells can switch their metabolism phenotypes in response to external stimuli for better survival. Taking into account the metabolic heterogeneity and plasticity of cancer cells, therapies targeting cancer metabolic dependency in principle can be made more effective.

## 1. Introduction

In the 1920s, Warburg and co-workers observed that in the presence of oxygen, rat liver carcinoma tissues have an approximately ten-fold increase in glucose to lactate conversion as compared to normal tissues [1]. This enhanced glycolysis exhibited by cancer cells under aerobic conditions is now referred to as the ‘Warburg effect’ or aerobic glycolysis. Warburg hypothesized that the enhanced glycolysis in cancer cells was due to the damage of mitochondrial respiration [2]. Upregulation of glucose transporters and glycolytic enzymes in rapidly growing tumor cells has been well documented since then [3,4]. However, the lack of evidence showing the mitochondrial defects in several cancer models has gradually weakened this hypothesis. Nonetheless, the Warburg effect was regarded as the dominant metabolic phenotype in cancer.

**Figure 1:**
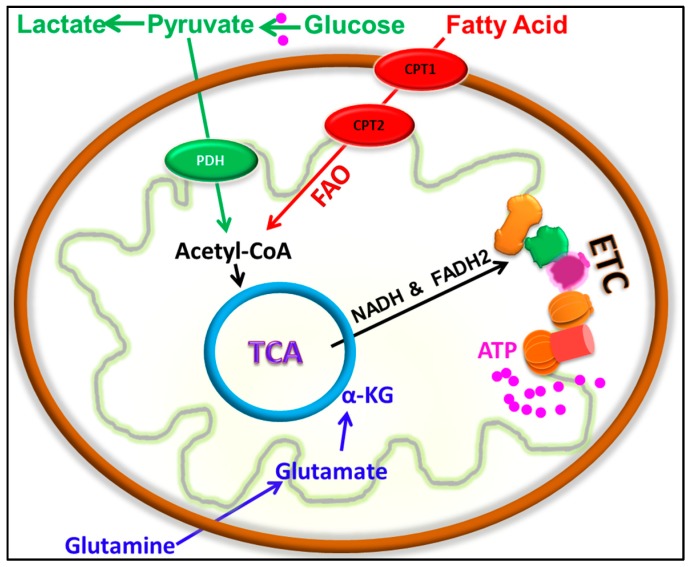
Major sources of mitochondrial energy pathways. Glucose, fatty acids, and glutamine are the major energy sources that support the tricarboxylic acid (TCA) cycle to generate ATP from the mitochondrial ETC.

Advances in the study of cancer metabolism over the last decades have changed our understanding on the effects of glycolysis and oxidative phosphorylation (OXPHOS) in cancer [4,5,6,7,8]. Increasing experimental evidence maintains a critical role for actively functional mitochondria in tumorigenesis, metastasis, cancer stemness, and therapy resistance [4,7,9,10,11,12,13]. Notably, mitochondria in cancer cells can utilize a broad range of metabolic pathways such as glucose oxidation, fatty acid β-oxidation (FAO) and glutamine oxidation to fuel the electron transport chain (ETC) for ATP production (Figure 1). For example, multiple studies showed that fatty acid can serve as a major energy source for triple negative breast cancer (TNBC) [10,14] and epithelial ovarian cancer [15]. In addition, combined mtDNA and whole-genome sequencing indicates that chromophobe renal cell carcinoma (chRCC) exhibits increased utilization of OXPHOS for ATP production relative to normal kidney [16]. Similarly, glutamine oxidation, usually driven by the oncogene MYC [17,18], also plays a critical role in energy production and promoting tumor growth in multiple cancer types [19]. It is important to note that the metabolic phenotype is not necessarily uniform across different types of tumors or even different tumors of the same type [11,13,20,21]. Due to the enhanced understanding of the importance and variability of cancer metabolism, metabolic reprogramming has attained the status of a hallmark of cancer [5,6].

In this review, we started by discussing the regulatory roles of mitochondria in determining tumor properties. Then, we review recent experimental studies towards elucidating the coupling of metabolic activities with tumor metastasis and cancer cell stemness. This experimental evidence supports the significance of OXPHOS and a hybrid (glycolysis and OXPHOS) metabolic phenotype in the subtypes of tumors. The hybrid metabolic state can provide metabolic plasticity for tumor cells to survive under different microenvironments to support tumor metastasis and therapy-resistance. Understanding the metabolic plasticity of individual tumors can help to design tumor-specific therapies including metabolic modulators to prevent the hybrid metabolic status and to sensitize tumor cells.

## 2. Retrograde Regulation of Tumor Properties by Mitochondria

**Figure 2:**
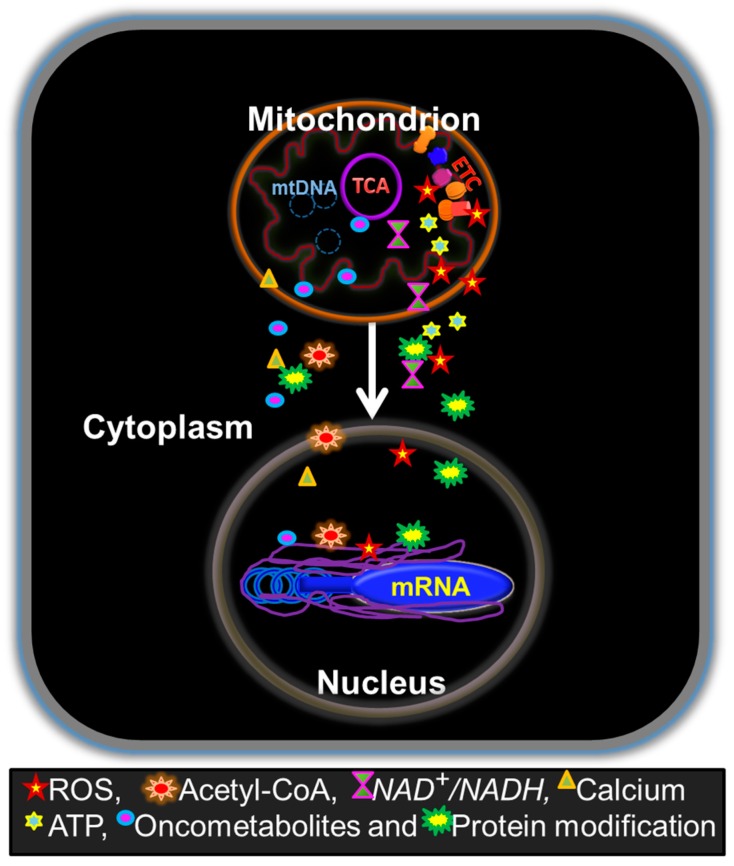
Schematic illustration of mitochondrial retrograde signaling. The illustration represents the major substrates and selected second messengers derived from mitochondrial function that play major roles in mitochondrial retrograde signaling. Retrograde signaling regulates the nuclear genome and transcriptional regulation as well as affects the posttranslational modification of proteins. Mitochondrial signals include but are not limited to ROS [63,64], Acetyl-CoA [10,65], NAD+/NADH ratio [66,67], calcium [68,69], ATP [10,70], and oncometabolites [71,72,73,74]. Availability of these regulators can be modulated by various mitochondrial properties including, mtDNA mutations, TCA activity, ETC function, and mitochondrial membrane potential. Mitochondrial signals can directly affect the nuclear genome by DNA mutation, histone modification, substrate availability etc. c-Src [10], MAPK [46], AMPK [75], PARPs [76,77], and SIRT1 [66,77] are examples of proteins that can be modified by the mitochondrial signaling. These protein modifications can further influence other protein targets and the nuclear genome.

Mitochondria contain their own genome, mitochondrial DNAs (mtDNAs), which encode mitochondrial respiratory chain complexes. In spite of the existence of mtDNAs, more than 98% of mitochondrial proteins are encoded by the nucleus genome [22], indicating obvious crosstalk between mitochondria and nucleus. Indeed, there is nucleus-to-mitochondria anterograde signaling and mitochondria-to-nucleus retrograde signaling [23]. Nuclear genomes have a dominant role in regulating replication and expression of mtDNAs [24], mitochondrial biogenesis [25], and metabolic activities. Mitochondria-to-nucleus retrograde signaling was first described in yeast [26] and later found in various organisms. This signaling governs the communication between mitochondria and the nucleus under various physiological and pathological conditions [27,28,29]. The mitochondria-to-nucleus retrograde signaling can be triggered by alterations in mtDNA copy numbers, mtDNA mutations, defects of mitochondrial respiratory chain complexes and also a change in mitochondrial reactive oxygen species (mtROS) levels [23]. Such retrograde signaling can adjust nuclear gene expressions for metabolic reconfiguration in response to these altered mitochondrial activities (Figure 2). Initial evidence for the importance of mitochondria in tumorigenesis was obtained by mtDNA depletion studies. In the 1990s, King and Attardi showed that the mtDNA in human cells can be depleted by exposing the cells to low concentrations of ethidium bromide and consequently the OXPHOS activity in the cells was repressed [30]. Initial observations from Hayashi et al., showed that the tumorigenicity of HeLa cells was lost after depletion of mtDNAs and recovered after reintroduction of mtDNAs [31]. Further studies in different cell models including ovarian, cervical carcinoma and osteogenic sarcoma showed that mtDNA-depleted cells are either poorly tumorigenic or non-tumorigenic [32]. In addition, the mtDNA-depleted brain and breast tumor cells exhibited impaired abilities to grow in an anchorage-independent manner and had increased sensitivity to cytotoxic drugs [33]. Recently, it has been shown that in melanoma and mammary carcinoma, tumor cells lacking mtDNAs, can only form tumors after acquiring mtDNAs from the host cells, which further validates the essential role of mitochondria in tumorigenesis [34]. In summary, mtDNA depletion studies from the 1990s onward have indicated the significance of mitochondrial integrity in tumorigenicity. 

Results from these mtDNA depletion studies gave rise to the idea of generating transmitochondrial cybrid models (comparing different mitochondria under a common nuclear background) to understand the functional significance of mtDNA variations [10,35,36,37,38,39,40,41,42]. Using cybrid technology, Ishikawa et al., published a pioneering study which showed that reactive oxygen species (ROS) induced mtDNA mutations contribute to tumor metastatic potential [35]. Several labs including ours also used the cybrid technology to demonstrate the critical effect of mitochondria-nuclear crosstalk in regulating tumor properties in multiple cancer types [10,35,36,37,38,39,40,41,42,43,44,45]. For example, by using cybrid models, we showed that mitochondrial FAO affects the autophosphorylation of the oncoprotein c-Src in TNBC. Inhibition of FAO almost completely aborts c-Src phosphorylation and suppresses tumorigenic and migratory properties of TNBC [10]. Other proteins like Calcineurin, PKC, CamKIV, JNK, and MAPK are also regulated by alteration of mitochondrial functions [46,47,48,49]. A review on the significance of cybrid models in cancer and underlying technical aspects has previously been published by our group [37,39]. Consistent with the aforementioned mtDNA-depletion results, studies using transmitochondrial cybrid models suggest a causal role of anomalously functioning mitochondria in tumorigenesis.

Anomalously functioning mitochondria in cancer can also result from mtDNA defects, such as mtDNA mutations that are mostly heteroplasmic [50] and mtDNA copy number reduction [51]. Several studies reported the association of mtDNA sequence variations and heteroplasmy in multiple cancer models [20,50,52,53,54,55,56]. Many mtDNA variations that affect mitochondrial ETC function have been identified with potential significance in tumor properties. Experimental approaches using breeding of multiple mice strains that contain different mtDNA variations provided in vivo experimental support for the role of mtDNA in tumor properties [57,58]. However, considering the heterogeneity of most of the tumors, multiple copies of mtDNA in single cells, and mtDNA selection by frequent fission or fusion of mitochondria, the contribution of an individual mtDNA variation to tumor progression is difficult to confirm. Further analysis using functional studies, haplotype analysis and extensive single cell sequencing are necessary to understand the role of individual mtDNA variations in tumor progression.

Mitochondria can communicate their changing metabolic states to the nucleus by retrograde signaling using various mediators (Figure 2). For example, a Ca^2 +^/Calcineurin (Cn) [59] signaling can activate multiple oncogenic factors including AKT and PI3K and upregulate glucose transporters GLUT1 and GLUT4 and glycolytic enzymes, thus shifting cell metabolism to glycolysis [23,60]. High glycolytic activity can further activate RAS through fructose-1,6-bisphosphate and reciprocally RAS can simulate glycolysis, thus forming a positive feedback loop [61]. Metabolic stress, such as glucose deprivation, can also drive KRAS mutations and the upregulation GLUT1, and consequently support cancer cells’ survival [62].

In addition to the altered mitochondrial function, abnormal accumulation of metabolites can also facilitate malignancy and such metabolites are referred to as oncometabolites [73,78,79]. Accumulation of oncometabolites is usually due to the mutations in genes encoding metabolic enzymes in the mitochondrial TCA cycle. For example, the loss-of-function mutation of fumarate hydratase (FH) lead to an accumulation of fumarate and this accumulation increases the metastatic potential and aggressiveness of renal cancer cells; this occurs by activating the epithelial-to-mesenchymal transition (EMT) through repression of miR-200 [80]. Similarly, loss of the mitochondrial tumor suppressor succinate dehydrogenase (SDH) causes the accumulation of succinate, which then promotes metastatic properties via the stabilization of hypoxia-inducible factor-1 alpha (HIF-1α) and consequently the activation of HIF-dependent pathways [81]. Another mitochondrial oncometabolite generated by the TCA cycle is d-2-hydroxyglutarate (d-2HG) due to the gain-of-function mutation of isocitrate dehydrogenase (IDH). IDH mutation is commonly observed in glioma, glioblastoma, and acute myeloid leukemia (AML) [82,83]. Accumulation of d-2HG inhibits 5-methylcytosine (5mC) hydroxylase TET2 activity and leads to a global DNA hypermethylation that impairs hematopoietic differentiation in AML and gliomas [74,84]. In addition, accumulation of d-2HG represses prolyl-hydroxylation of collagen that results in a defective basement membrane that might contribute to glioma progression [74]. Moreover, high levels of d-2HG induce an EMT-like phenotype via direct upregulation of ZEB1 expression by promoting the H3K4 trimethylation of the promoter region of ZEB1 in colorectal cancer cells [72]. Finally, in addition to mutations of metabolic enzymes in the TCA cycle, accumulation of the metabolic products of dihydropyrimidine dehydrogenase (DPYD), a rate-limiting enzyme in pyrimidine degradation, has been shown to be essential for the EMT in benign breast epithelium HMLE cells. Overexpression of DPYD can in fact accelerate the EMT [85].

In summary, mitochondria-to-nucleus retrograde signaling in cancer may be an adaption mechanism by which altered mitochondrial function modulates nuclear gene expressions towards tumorigenesis and invasiveness. This is a somewhat different role for neoplastic mitochondria than originally proposed by Warburg.

## 3. Significance of Mitochondrial Biogenesis and Respiration in EMT and Metastasis

Metastasis accounts for most of cancer-related deaths [86]. Metabolic activities in metastasized cancer cells are usually reprogrammed to support and promote their migratory and invasive capacities [87,88]. A variety of studies have shown that metastasis associates with an enhanced mitochondrial respiration and biogenesis activity and inhibition of OXPHOS suppresses metastasis in breast and cervical cancer. For example, the metastatic propensity of TNBC MDA231 cells is largely dependent on their mitochondrial FAO activity and pharmacologic inhibition of FAO significantly represses in vivo tumor growth potential [10,14]. Highly metastatic mouse breast cancer 4T1 cells, that are usually used for the study of stage IV human breast cancer [89], exhibit both enhanced glycolytic and increased OXPHOS activities as compared to non-metastatic isogenic 67NR cells [90]. Consistently, another study shows that the circulating tumor cells (CTCs) exhibit significantly higher mitochondrial respiration and biogenesis activity compared to both the primary tumors from 4T1 cells and its lung metastases [9]. The enhanced OXPHOS in 4T1 cells is modulated by peroxisome proliferator-activated receptor gamma coactivator 1 (PGC-1)α, whose expression is associated with the EMT program in vivo, and distant metastasis and poor prognosis of patients with invasive ductal carcinomas [9]. Notably, these CTCs derived from 4T1 cells showed no decrease in their glycolytic activity, indicating a hybrid metabolic phenotype [8]. Such a hybrid metabolic phenotype has also been observed in superinvasive human cervical carcinoma and melanoma cells. Increased mitochondrial superoxide production by either ETC overload or partial ETC inhibition promotes the metastatic property and clonogenicity of SiHa human cervix squamous cell carcinoma cells [12]. Both in vitro selection of superinvasive SiHa-F3 cells and in vivo selection of supermetastatic B16-M4b cells show increased OXPHOS or production of TCA intermediates without an observable change in their lactate production rates [12]. Taken together, these results suggest an important role for mitochondrial biogenesis and respiration during metastasis and indicate that increased metastatic potentials might be specifically associated with a hybrid glycolysis/OXPHOS phenotype.

Metastases of carcinoma cells are often facilitated by EMT, a transdifferentiation program by which epithelial cancer cells lose cell–cell adhesion and concomitantly acquire mesenchymal features of migration and invasion [91]. EMT has been shown to be coupled with metabolic reprogramming [92]. Enhanced mitochondrial biogenesis and respiration has been observed during EMT in breast, pancreatic, esophageal, and lung cancer. For example, CTC from the 4T1 mammary carcinoma, as mentioned before, exhibit increased mitochondrial biogenesis and respiration that is co-induced with EMT [9]. The mesenchymal subpopulation of HMLE cells exhibits a higher OXPHOS activity as compared to their epithelial counterpart [93]. The human pancreatic cancer PANC-1 cells undergoing TGFβ-1-induced EMT show strong increases in mitochondrial mass, mtDNA content, and ROS production [94]. In the esophageal squamous cell carcinoma (ESCC) cell line TE1, high mtDNA copy number and mitochondria bioenergetic function correlate with upregulation of EMT markers and tumor invasiveness [95]. TGFβ-1 induced EMT accompanies an increase of oxygen consumption and decrease of fatty acid synthesis via SNAIL1-mediated inhibition of ACC and FASN in NSCLC A549 cells [96]. Moreover, diversion of glucose to the TCA cycle, partially due to reduction in the PDK4 expression, is necessary for TGFβ-1 induced EMT in several NSCLC cell lines including A549 and HCC827. Inhibition of PDK4 alone can induce EMT [97].

Conversely, results from other studies tend to connect enhanced glycolytic activities with EMT and metastasis. Gaude et al., shows that downregulation of mitochondrial genes associates with EMT and poor prognosis across multiple cancer types by analyzing the patients’ data from the Cancer Genome Atlas (TCGA) [98]. Fast-growing solid tumors usually face a progressively hypoxic situation that can induce and stabilize HIF-1α. HIF-1α is a master regulator of glycolysis [99], and also a well-known EMT inducer by upregulating EMT transcription factors (EMT-TFs), such as SNAIL and TWIST [100,101], thus potentially connecting glycolysis with EMT. Overexpression of TWIST has been shown to increase glucose consumption and lactate production and decrease mitochondrial mass in MCF10A cells [102]. Since accumulation of lactate can strongly increase the protein levels of HIF-1α, there seems to be a positive feedback loop between HIF-1α and glycolysis. Another study shows that metabolic stress can activate AMP-activated protein kinase (AMPK), a master regulator of mitochondrial biogenesis and respiration, and AMPK activation blocks EMT by activating FOXO3a in 4T1 and PC-3 cells; consistently, silencing AMPK promotes EMT in these cell lines [103]. Increased activity of mitochondria complex I can repress tumor growth and metastasis partly through the regulation of NAD^+^/NADH redox balance in MDA-MB-435 and MDA-MB-231 cells [104].

**Figure 3:**
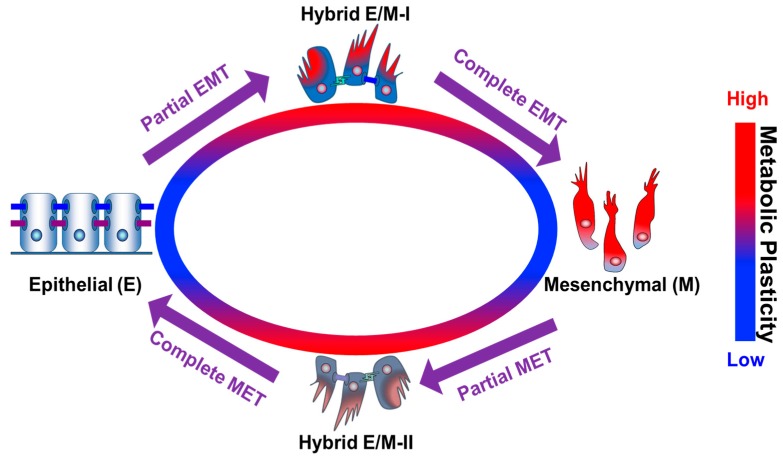
EMT and metabolic plasticity. As compared to the epithelial (E) and mesenchymal (M) phenotypes, the hybrid E/M phenotypes during EMT/MET may have higher metabolic plasticity.

At present, it appears that the association of enhanced mitochondrial respiration or increased glycolytic activity with EMT and metastasis may be context-dependent. In all cases, however, metastasis is strongly coupled to mitochondrial activity. The discrepancies in the association of metastasis with metabolism might be attributed to the different metastatic sites. For example, primary breast cancer 4T1 cells can metastasize into liver, lung, and bone and in general liver metastases exhibit higher glycolysis and lower mitochondrial respiration relative to lung and bone metastases [90]. To further elucidate the coupling between EMT and metabolism, a rigorous and quantitative assessment of cell phenotypes and tumor microenvironment in terms of EMT and metabolism is needed. Metastasis involves cycles of EMT and the reverse process, mesenchymal-to-epithelial transition (MET) [105], in which cancer cells can exhibit a broad spectrum of hybrid epithelial/mesenchymal (E/M) phenotypes that combine partial epithelial traits, cell–cell adhesion, and partial mesenchymal traits, migratory and invasive properties [106,107,108,109,110,111]. Cancer cells in all these states appear to be capable of using various metabolic pathways, such as glycolysis and OXPHOS, including glucose, fatty acid and glutamine oxidation, and their combinations for energy production and biomass synthesis. A more accurate characterization of both EMT and metabolism phenotypes can contribute to a better understanding of their connections. Indeed, two EMT scoring methods [112,113] and an AMPK/HIF-1 signature [8] have been developed to evaluate the EMT status and OXPHOS/glycolysis activity respectively based on gene expression data across cancer types. Future work integrating both gene expression data and metabolite abundance may contribute to a better understanding of the EMT-metabolism interplay. Particular attention should be paid to the potential coupling between hybrid epithelial/mesenchymal (E/M) and hybrid glycolysis/OXPHOS phenotypes (Figure 3) [9,12,90], since these hybrid phenotypes have been proposed as ‘chief instigators’ of metastases [8,9,12,90,106,107,108,109,110,111,114,115]. Considering the aforementioned experimental work, one hypothesis regarding the coupling of EMT with metabolic activity is that high glycolytic activity promotes partial EMT [98], by which epithelial cells can transition into a hybrid E/M phenotype (Figure 3). Once induced, the hybrid E/M cells might upregulate their mitochondrial activity for more effective ATP production to facilitate their migration and invasion, as suggested by the study of 4T1 CTCs [9] since the CTC clusters are proposed to be hybrid E/M cells [114]. OXPHOS activity might stabilize the epithelial phenotype and repress partial EMT. Notably, the association of cell phenotypes—epithelial, hybrid E/M, and mesenchymal—with metabolism phenotypes needs not to be the same as the association of the processes—partial EMT, complete EMT, partial MET and complete MET—with metabolic activities (Figure 3). The hypothesis proposed here of course requires rigorous experimental tests both in vitro and in vivo.

## 4. Mitochondrial Dependency in Cancer Stemness

Tumor relapse is believed to be initiated by therapy-resistant cancer stem cells (CSCs), which are poorly differentiated and have the capacity for self-renewal and the generation of more differentiated progeny [116]. Increased mitochondrial mass, membrane potential and enhanced mitochondrial respiration have been widely observed in CSCs across multiple types of cancer. Due to the dependency of CSCs on mitochondrial activity, it seems that CSCs are more vulnerable to therapies targeting mitochondrial respiration [117,118,119,120]. For example, compared to the differentiated progeny that depends primarily on glycolysis, breast cancer stem cells (BCSCs) derived from MCF7, T47D, MDA-MB-231, and SUM159PT cells show a reliance on OXPHOS, characterized by more glucose consumption, less lactate production, and higher ATP content [21]. Consistently, suppression of OXPHOS by the drug XCT790, a well-established inhibitor of the estrogen-related receptor α (ERRα)–PGC-1α signaling pathway, or by the drug doxycycline, a FDA-approved antibiotic, decreases the survival and propagation of MCF7 CSCs [120]. Another study showed that increased mitochondrial mass, confers stem-like traits of MDA-MB-231 and MCF7 cells and enables their resistance to paclitaxel [121]. Indeed, high mitochondrial mass has been indicated as a metabolic biomarker for the anabolic CSCs in MCF7 cells [122]. In addition to breast cancer, the CSCs isolated from epithelial ovarian cancer patients also show enhanced OXPHOS activity [15]. These CSCs underwent apoptosis when the mitochondrial respiratory chain was repressed. Pancreatic CSCs show more dependency on OXPHOS while non-CSCs are more glycolytic, which may explain why the drug metformin, an inhibitor of mitochondrial ETC complex I, target pancreatic CSCs but not the non-CSCs [117]. Moreover, after KRas ablation, the surviving pancreatic ductal adenocarcinoma (PDAC) cells, that account for tumor relapse, rely on mitochondrial respiration and are highly sensitive to OXPHOS inhibitors [123]. Glioblastoma CSCs rely on OXPHOS and repressing OXPHOS but not glycolysis abolishes their tumorigenicity [124]. Increased mitochondrial biogenesis and elevated OXPHOS contribute to the resistance of melanoma to BRAF inhibitors [125]. Consistently, OXPHOS inhibitors decrease the prevalence of BRAF inhibitor-resistant slow-cycling melanoma cells [119]. Similarly, inhibition of OXPHOS can selectively eradicate quiescent leukemia stem cells via BCL-2 inhibition [126]. All these results support a critical role of the mitochondrial respiration and biogenesis for the survival and propagation of CSCs. Note that these findings are consistent with the correlation between cells that undergo (partial) EMT and cells that exhibit stem-like properties, if we assume as discussed above that in most cases EMT leads to enhanced OXPHOS.

CSCs exhibit elevated rates of oxygen consumption and ROS production as compared to the differentiated cells [13]. CSCs also have a more powerful antioxidant capabilities as compared to their differentiated progeny [127]. It has been shown that CSCs can maintain their ROS levels lower than those in their progeny and such moderate ROS levels in CSCs enable their tumorigenic property and radioresistance [128]. For example, melanoma cells with high PGC-1α expression, driven by the melanocyte lineage-specification transcription factor (MITF), exhibit enhanced mitochondrial respiration and ROS detoxification capacity, which enable these melanoma cells to survive under oxidative stress conditions [127].

In cancer there is never “one size fits all” and notably, there are also studies showing that CSCs prefer glycolysis. For example, BCSCs isolated from human breast cancer patients show a preference for glycolysis as characterized by increased activities of glycolytic enzymes such as lactate dehydrogenase (LDH) and pyruvate kinase M2 isoform (PKM2) [129]; this is different than the observation of BCSCs derived from cell lines as discussed before. Use of 2-deoxyglucose (2-DG), an inhibitor for glycolysis, can inhibit the proliferation of these BCSCs. The ovarian cancer spheroid cells with stem-like behaviors exhibit an increase in their glycolytic flux as compared to their parental cells [130]. Glioblastoma cells with stem-like properties exhibit a preference for glycolysis for ATP generation and maintain their stemness under hypoxia [131].

These different metabolic patterns of CSCs might be due to distinctive molecular characteristics. For example, two subpopulations of BCSCs, one is characterized by ALDH*^high^* and the other is characterized by CD44*^high^*/CD24*^low^*, have been observed. These two subpopulations might be interconvertible [132]. Another study shows that the EMT CSCs characterized by low expression of epithelial specific antigen (ESA), are quiescent, and exhibit lower mitochondrial mass, membrane potential, oxygen consumption, and ROS production as compared to the epithelial CSCs characterized by high ESA expression in several head and neck squamous cell carcinoma (HNSCC) cell lines [133]. Recently, a subpopulation of normal human breast stem cells (BSCs), exhibiting hybrid ALDH*^high^*/CD44*^high^*/CD24*^low^* expression, has been characterized and such ALDH*^high^*/CD44*^high^*/CD24*^low^* BSCs have the highest mammosphere formation capacity at the single cell level as compared to apparently more restrictive ALDH*^high^* and CD44*^high^*/CD24*^low^* BSCs [134], hinting that the extent of being stem-like varies among individual subpopulations of stem cells. It seems therefore that the metabolic activities of CSCs at least partially depend on their exact molecular characteristics. Nonetheless, the survival and propagation of CSCs is strongly affected by their mitochondrial function. Future work to characterize the molecular characteristics of different CSCs and possibly varying degree of stemness might contribute to a better understanding of the stemness-metabolism interplay.

## 5. Emergence of a Hybrid Metabolic Phenotype in Cancer Cells

**Figure 4:**
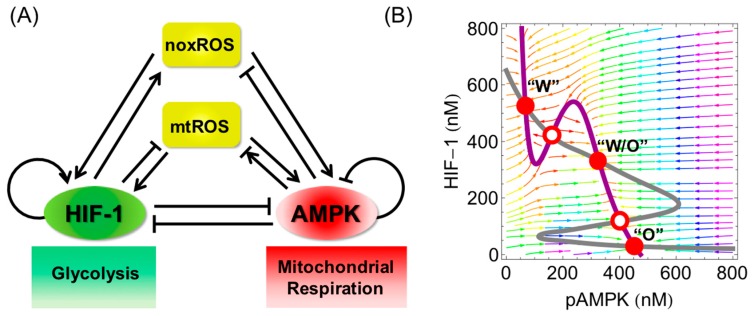
Modeling the interplay between glycolysis and OXPHOS in cancer. (**A**) The AMPK:HIF-1:ROS regulatory circuit. The arrows represent the excitatory regulations and the bar-headed arrows represent inhibitory regulations; (**B**) Nullclines and steady states in the phase space of pAMPK and HIF-1. The AMPK activity is represented by the level of phosphorylated AMPK (pAMPK) at threonine-172 of the α subunit. The gray line represents the nullcline of dh/dt=0, where h represents the levels of HIF-1. The purple line represents the nullcline of dA/dt=0, where A represents the levels of pAMPK. The intersections of these two nullclines represent the steady states of the regulatory circuit shown in (**A**). The arrow denotes the direction of motion in the vector field. The steady state corresponding to various initial conditions can be identified following the arrows. The red solid dots represent the stable steady states, i.e., stable metabolic phenotypes. The red hollow dots represent the unstable steady states, i.e., unstable metabolic phenotypes. “W” represents the Warburg state, i.e., aerobic glycolysis. “O” represents the OXPHOS state. “W/O” represents the hybrid glycolysis/OXPHOS state. More details of the model can be found in [8].

Despite the advances in understanding of the significance of mitochondria in tumorigenesis, metastasis, and stemness, it is still elusive as to how different metabolic phenotypes are orchestrated in cancer cells. To shed light upon the interplay between glycolysis and OXPHOS, we used a systems biology approach to develop a mathematical model integrating AMPK, a master regulator for mitochondrial biogenesis and respiration, HIF-1, a master regulator for glycolysis, and ROS including both mtROS and cytosol ROS (noxROS) since ROS plays a critical role in mediating the interplay between AMPK and HIF-1 (Figure 4A) [8]. The AMPK:HIF-1:ROS circuit predicts that cancer cells can acquire three stable phenotypes, a glycolysis phenotype characterized by high HIF-1 and low AMPK activities, an OXPHOS phenotype characterized by low HIF-1 and high AMPK activities and a hybrid glycolysis/OXPHOS metabolic phenotype, characterized by both high HIF-1 and high AMPK activities (Figure 4B). The model further shows that the hybrid metabolic phenotype can be promoted by elevated production rates of mtROS, stabilization of HIF-1 and regulation of oncogenes such as MYC and c-SRC [8].

The hybrid glycolysis/OXPHOS metabolic phenotype can provide several advantages to cancer cells as indicated by both the modeling and experimental studies. First, the hybrid metabolic phenotype endows cancer cells with the flexibility to utilize various kinds of available nutrients, such as glucose, fatty acid and glutamine, to satisfy the bioenergetic and biosynthetic needs for tumor development in different microenvironments. Second, cancer cells in the hybrid metabolic phenotype can efficiently produce energy by both OXPHOS and glycolysis. Meanwhile, the byproducts from glycolysis, such as lactate and pyruvate, can be utilized for biomass synthesis to facilitate cell proliferation. Third, since the hybrid metabolic phenotype maintains ROS at a moderate level, cancer cells in the hybrid metabolic phenotype can benefit from moderate ROS-mediated stress response and mutagenic events that stimulate tumorigenesis and metastasis, and avoid the detrimental effects of excessive ROS [12,35,135]. Fourth, the hybrid metabolic phenotype may be specifically associated with metastasis [9,10,12,90,134], as discussed before. Fifth, the hybrid phenotype might promote the therapy-resistance of cancer cells. For example, during metformin treatment, the resistant pancreatic CSCs emerge with an intermediate glycolytic/respiratory phenotype [117]. Collectively, cancer cells in the hybrid metabolic phenotype have a plethora of benefits over cells using only glycolysis or OXPHOS.

## 6. Cancer Mitochondrial Respiration Driven by Cancer-Associated Fibroblasts

It is important to recognize that metabolic reprogramming, as a hallmark of cancer, involves not only the cancer cells. Instead, cancer-associated fibroblasts (CAFs), stromal cells which often dominate the tumor microenvironment, are prone to glycolysis by which CAFs provide energy-rich metabolites to fuel the mitochondrial respiration and anabolic metabolism of cancer cells [136,137]. This coupled metabolic pattern between cancer cells and surrounding CAFs is sometimes referred to as the “reverse Warburg effect” [138,139,140,141]. With an elevated production of ROS, cancer cells can secrete ROS into surrounding microenvironment that pushes the CAFs to utilize aerobic glycolysis and produce high-energy metabolic intermediates, such as pyruvate, ketone bodies, lactate, and fatty acid. These metabolic intermediates can be transported to the cancer cells and fuel mitochondrial respiration for efficient ATP production [141,142]. The ROS secreted by cancer cells can reduce the production of caveolin-1 (Cav-1), an important structural protein that is involved in endocytosis and vesicular transport. Loss of Cav-1 in CAFs results in additional ROS production in cancer cells, thus forming a positive feedback for the oxidative stress on CAFs and consequently active mitochondrial respiration in cancer [143]. Indeed, loss of Cav-1 has been used as an independent biomarker for poor prognosis in various types of tumors [139,143]. The tumor-promoting effects of CAFs could be reversed by two inhibitors of glycolysis, 2-DG and dichloro-acetate (DCA) [141]. The aerobic glycolysis of CAFs results from the stabilization of HIF-1α following the downregulation of the IDH3α [144]. Consistently, IDH3α overexpression in CAFs greatly reduces the tumor-promoting effects of CAFs in vivo. In addition, high expression of mono-carboxylate transporter (MCT) 4, that is referred to as a ‘lactate shuttle’, modulates transportation of metabolic intermediates produced in CAFs to cancer cells and correlates with poor overall survival of TNBC patients [145,146]. Taken together, the mitochondrial respiration of cancer can be promoted by the surrounding glycolytic CAFs and targeting the glycolytic events or the transportation of metabolic intermediates could weaken the tumor-promoting effects of CAFs.

## 7. Therapies towards Targeting the Metabolic Dependency of Cancer Cells

Recent advancement in metabolic research has made it clear that the altered metabolism in cancer is not only a secondary effect due to the signaling regulation for growth and proliferation but also can be a primary cause for tumorigenic, metastatic, and stem-like events [5,6,23,60]. Since the metabolic dependency of tumors is heterogeneous, therapies targeting metabolism may not be uniformly effective [147]. For example, ketogenic diets, which are low in glucose and other carbohydrates but high in fats, can force cancer cells to utilize mitochondrial respiration. The ketogenic diets are supposed to cause more oxidative stress in cancer cells, thus sensitizing cancer to conventional radiation and chemotherapies [148]. However, for certain types of cancer such as TNBC that shows a high dependency on FAO [10,14], the ketogenic diet may worsen the tumor status. Another proposed therapeutic strategy is activation of pyruvate dehydrogenase (PDH). However, human NSCLC tumors exhibited enhanced PDH activity as compared to adjacent benign lung [20] and PDH activity is associated with EMT and drug resistance in NSCLC A549 and HCC827 cells [97]. In such scenario, PDH activation may not be therapeutically useful and malignancy may be promoted instead. Thus, due to the heterogeneity of cancer metabolism, the metabolic therapies should be selected according to the metabolic dependency of specific cancer cells.

The emergence of a hybrid glycolysis/OXPHOS metabolic phenotype, which might primarily account for tumor metastatic potential, stem-like property, and therapy-resistance, implies that proper blockade of both glycolysis and OXPHOS may be a more promising approach since it could potentially eliminate the metastatic plasticity of cancer. Indeed, dual inhibition of cancer metabolism by metformin, (inhibitor of ETC complex 1), and 2-DG, (glycolysis blocker) has shown good effects against tumor growth and metastasis across multiple preclinical cancer models [149]. The beneficial effects of this combination therapy is also explained by our modeling analysis that shows that it can effectively drive cancer cells out of the hybrid metabolic phenotype [8]. In addition, since cancer cells are capable of switching their metabolism phenotypes during treatment, therapies blocking the metabolic switch could potentially impair tumor viability [150]. Collectively, due to the enriched metabolic plasticity of cancer, future therapeutic strategies might consider targeting the hybrid glycolysis/OXPHOS phenotype and eliminating the metabolic phenotypic transition capability of cancer cells to improve cancer treatment outcome. The proposed strategies here need to be carefully evaluated combining both experimental and theoretical modeling efforts [8,151,152,153,154,155,156].

## Abbreviations

2-DG	2-deoxyglucose
5mC	5-methylcytosine
AML	acute myeloid leukemia
AMPK	AMP-activated protein kinase
BCSCs	breast cancer stem cells
BSCs	breast stem cells
CAFs	cancer-associated fibroblasts
Cav-1	caveolin-1
chRCC	chromophobe renal cell carcinoma
Cn	calcineurin
CSCs	cancer stem cells
CTCs	circulating tumor cells
d-2HG	d-2-hydroxyglutarate
DCA	dichloro-acetate
DPYD	dihydropyrimidine dehydrogenase
EMT	epithelial-to-mesenchymal transition
EMT-TFs	EMT transcription factors
ERRα	estrogen-related receptor α
ESA	epithelial specific antigen
ESCC	esophageal squamous cell carcinoma
ETC	electron transport chain
FAO	fatty acid β-oxidation
FH	fumarate hydratase
HIF-1α	hypoxia-inducible factor-1 alpha
HNSCC	head and neck squamous cell carcinoma
hybrid E/M	hybrid epithelial/mesenchymal
IDH	isocitrate dehydrogenase
LDH	lactate dehydrogenase
MET	mesenchymal-to-epithelial transition
MITF	melanocyte lineage-specification transcription factor
mtROS	mitochondrial reactive oxygen species
mtDNA	mitochondrial DNA
noxROS	NADPH oxidase-derived reactive oxygen species
OXPHOS	oxidative phosphorylation
PDAC	pancreatic ductal adenocarcinoma
PDH	pyruvate dehydrogenase
PGC-1	peroxisome proliferator-activated receptor gamma coactivator 1
PKM2	pyruvate kinase M2 isoform
ROS	reactive oxygen species
SDH	succinate dehydrogenase
TNBC	triple negative breast cancer
TCA	tricarboxylic acid
TCGA	The Cancer Genome Atlas
